# Late Adverse Events After Chimeric Antigen Receptor T-Cell Therapy for Patients With Aggressive B-Cell Non-Hodgkin Lymphoma

**DOI:** 10.1001/jamanetworkopen.2024.61683

**Published:** 2025-02-25

**Authors:** Lina Camacho-Arteaga, Gloria Iacoboni, Mi Kwon, Rebeca Bailén, Rafael Hernani, Ana Benzaquén, Lucía López-Corral, Estefania Pérez-López, Lina María Leguízamo-Martínez, Maria Calvo-Orteu, Manuel Guerreiro, Aitana Balaguer-Rosello, Carla Alonso-Martínez, Xavier Vidal, Pere Barba, Antònia Agustí

**Affiliations:** 1 Department of Clinical Pharmacology, University Hospital Vall d’Hebron, Barcelona, Spain; 2 Department of Pharmacology, Therapeutics and Toxicology, Universitat Autònoma de Barcelona, Bellaterra, Spain; 3 Clinical Pharmacology Research Group, Vall d’Hebron Institut de Recerca (VHIR), Barcelona, Spain; 4 Department of Hematology, University Hospital Vall d’Hebron, Barcelona, Spain; 5 Experimental Hematology, Vall d’Hebron Institute of Oncology (VHIO), Barcelona, Spain; 6 Department of Medicine, Universitat Autònoma de Barcelona, Bellaterra, Spain; 7 Department of Hematology, Hospital General Universitario Gregorio Marañón, Madrid, Spain; 8 Institute of Health Resarch Gregorio Marañón, Madrid, Spain; 9 Department of Medicine, Universidad Complutense de Madrid, Madrid, Spain; 10 Hematology Department, Hospital Clínico Universitario, INCLIVA Research Institute Valencia, Spain; 11 Department of Hematology, Hospital Universitario de Salamanca, IBSAL, CIBERONC, Salamanca, Spain; 12 Department of Clinical Pharmacology, Area Medicament, Hospital Clinic of Barcelona, Barcelona, Spain; 13 Clinical Pharmacology, Institut d’Investigacions Biomèdiques August Pi i Sunyer (IDIBAPS), Barcelona, Spain; 14 Department of Hematology, Hospital Universitari i Politecnic La Fe, Valencia, Spain; 15 CIBERONC, Instituto Carlos III, Madrid, Spain; 16 Department of Pharmacy, Vall D’Hebron University Hospital, Barcelona, Spain

## Abstract

**Importance:**

Acute adverse events (AEs) after chimeric antigen receptor (CAR) T-cell infusion are well documented, but less information is available regarding the long-term toxic effects.

**Objective:**

To assess the occurrence of late AEs for adult patients with large B-cell lymphoma (LBCL) treated with commercially available CD19-targeted CAR T cells.

**Design, Setting, and Participants:**

A prospective, observational, clinical practice cohort study was conducted from September 1, 2018, to December 31, 2022, among 172 adult patients in 6 Spanish hospitals who received CD19-targeted CAR T-cell therapy for relapsed or refractory LBCL and survived at least 3 months after infusion, without subsequent antilymphoma therapy.

**Exposure:**

Treatment with tisagenlecleucel or axicabtagene ciloleucel.

**Main Outcomes and Measures:**

Data on any late AEs occurring in this patient population were collected until the patients received new antilymphoma therapy, were lost to follow-up, died, or reached 24 months after infusion, whichever occurred first. Data collection for each patient started at the third month after infusion and included new-onset AEs, as well as persistent AEs that started earlier but were still ongoing at that time point.

**Results:**

The study enrolled 172 patients (mean [SD] age, 58.5 [13.7] years; 101 men [58.7%]), of whom 135 (78.5%) experienced at least 1 late AE of any grade. Infections were the late AEs with the highest incidence (5.6 per 100 person-months [95% CI, 4.5-7.0 per 100 person-months]), followed by neutropenia (3.6 per 100 person-months [95% CI, 2.9-4.5 per 100 person-months]) and thrombocytopenia (2.2 per 100 person-months [95% CI, 1.7-3.0 per 100 person-months]). The incidence of infectious episodes remained stable during the whole study period, while cytopenias decreased beyond 6 months after infusion. All cases of nonrelapse-related mortality were due to infections (COVID-19 pneumonia in 3 patients and sepsis or bacterial pneumonia in 4 patients). Twenty-three patients (13.4%) experienced 27 dermatologic AEs, all mild, with most of them (88.9% [24 of 27]) starting beyond 3 months after infusion. Fifteen neurologic AEs were reported in 15 patients (8.7%), and 10 patients (5.8%) developed 13 cardiovascular AEs. Five secondary neoplasms were reported in 4 patients (2.3%), with no cases of T-cell malignant neoplasms.

**Conclusions and Relevance:**

This cohort study suggests that CAR T-cell therapy has a favorable safety profile. However, continuous follow-up of patients is needed, as serious AEs can occur years after infusion.

## Key Points

**Question:**

What is the late safety profile for adult patients with aggressive B-cell non-Hodgkin lymphoma treated with commercially available CD19-targeted chimeric antigen receptor (CAR) T cells?

**Findings:**

In a cohort study of 172 patients who survived at least 3 months after the CAR T-cell infusion without subsequent antilymphoma therapy, infections and cytopenias were the most common late adverse events after that time point; other late complications included neurologic, cardiovascular, and dermatologic events. Five secondary malignant neoplasms were reported, with no cases of T-cell malignant neoplasms in the patient population.

**Meaning:**

This study suggests that CAR T-cell therapy has a favorable long-term safety profile.

## Teaser

This cohort study assesses the occurrence of late adverse events for adult patients with aggressive large B-cell non-Hodgkin lymphoma treated with commercially available CD19-targeted chimeric antigen receptor (CAR) T cells.

## Introduction

Chimeric antigen receptor (CAR) T-cell therapy has improved the outcome of patients with relapsed or refractory aggressive large B-cell lymphoma (LBCL), providing high response rates and prolonged survival to a significant proportion of patients. However, this treatment modality has a specific toxic effect profile. Short-term adverse events (AEs), including cytokine release syndrome (CRS), immune effector cell–associated neurotoxicity syndrome (ICANS), and early immune effector cell–associated hematotoxicity, have been widely described in pivotal trials and clinical practice data.^1,2,3,4,5,6,7,8,9,10,11^ However, the incidence and characteristics of late AEs, occurring beyond the first weeks after infusion, are not as well documented.^12,13,14^ A retrospective study analyzed late AEs among patients treated with CD19 CAR T cells in phase 1 and 2 precommercialization clinical trials, and identified hypogammaglobulinemia and infections as the most frequent long-term AEs. Less common, but clinically relevant, events described in this study included subsequent malignant neoplasms, late-onset neurologic and psychiatric disorders, immune-related events, and graft-vs-host disease.^12^ Other organ toxic effects described after CAR T-cell infusion include cardiac^15^ and kidney events.^16^ Recently, the US Food and Drug Administration (FDA) added a boxed warning to CAR T-cell therapies after T-cell lymphoma reports were issued for patients previously treated with CAR T cells; however, the frequency of these events seems to be extremely low and, in most cases, not directly associated with prior CAR T-cell therapy.^17,18,19^

The severity of these short-term and long-term toxic effects is heterogeneous, ranging from mild events that resolve spontaneously to severe or life-threatening complications. The risk of nonrelapse mortality (NRM) after CAR T-cell therapy among patients with relapsed or refractory LBCL is approximately 5% to 10%, mainly due to infections.^7,8,9,20,21^ However, this risk might be higher for patients with other disease entities, such as mantle cell lymphoma and acute lymphoblastic leukemia.^22,23,24^

Most studies reporting long-term AEs after CAR T-cell therapy were retrospective and focused mainly on describing the effectiveness and early toxic effects. Others were restricted to patients included in clinical trials or were focused exclusively on a specific type of organ toxic effect,^15,16^ harboring the potential risk of underrepresenting other long-term AEs. In addition, patients undergoing CAR T-cell treatment are usually treated in highly experienced infusing centers and return to the referring center 4 to 12 weeks after the infusion. Hence, as most of the long-term toxic effect events occur beyond this time point, collecting this information retrospectively can be challenging. Given the scarce available information on long-term AEs associated with commercial CAR T-cell therapy, this study aimed to prospectively collect any late AEs occurring in adult patients with aggressive LBCL who received CD19-targeted CAR T cells in the clinical practice setting.

## Methods

### Study Design

A prospective, observational, multicenter study was carried out in 6 Spanish CAR T-cell centers from September 1, 2018, to December 31, 2022. All adult patients with a diagnosis of aggressive LBCL treated with commercial, CD19-directed, CAR T-cell products (tisagenlecleucel and axicabtagene ciloleucel) in the third-line or later setting, who were alive at 3 months after infusion and had not received any further treatment for their disease, were included. The protocol and informed written consent were approved by the Clinical Research Ethics Committee of Vall d’Hebron University Hospital. The study was performed in accordance with the Declaration of Helsinki.^25^ The study followed the Strengthening the Reporting of Observational Studies in Epidemiology (STROBE) reporting guideline.

Patients meeting the inclusion criteria were enrolled into the study at 3 months after infusion, and their baseline information was then collected. This time point was selected based on the expected period for a complete resolution of the most common acute toxic effects (CRS and ICANS).^14^ Ongoing AEs at time of study entry and new AEs were prospectively registered in an electronic REDCap database. All patients had 6 scheduled study visits at 3, 6, 9, 12, 18, and 24 months after CAR T-cell infusion. At these time points, information on new AEs and persistent AEs (which had started before 3 months after the infusion) was obtained. For each AE, the start and end date, severity, treatment, and outcome were collected. Data regarding laboratory parameters and disease assessment via positron emission tomographic and computed tomographic (PET-CT) scan were also registered. Follow-up ended if the patient received new antilymphoma therapy, was lost to follow-up, died, or reached 24 months after infusion, whichever occurred first. The last visit registered in the REDCap database captured the patient and disease status and the reason for end of follow-up.

### Patients

All patients had been previously evaluated by the national centralized multidisciplinary committee for CAR T-cell therapy, and the indication was approved based on homogeneous criteria from the Ministry of Health, Consumer Affairs and Social Welfare.^26^ Lymphodepleting chemotherapy included fludarabine and cyclophosphamide for all patients, in accordance with manufacturers’ recommendations. Management of CRS and ICANS followed local practice guidelines, based largely on the Spanish national guidelines.^27^ Disease evaluation was carried out locally using PET-CT scans at postinfusion months 1, 3, 6, 12, and 18, and graded according to Lugano criteria^28^; additional imaging was performed if progressive disease was suspected at any time after CAR T-cell infusion. During the first month after the CAR T-cell infusion, follow-up was performed in person. After this period, patients returned to their referral center and follow-up was continued remotely.

### Variable Definition and End Points

Patient characteristics and comorbidities, as well as data on the lymphoma and CAR T-cell product, were retrieved from their medical records. Post-CAR T-cell infusion AEs were classified using the *International Statistical Classification of Diseases and Related Health Problems, 10th Revision*,^29^ and their severity was graded using the Common Terminology Criteria for Adverse Events, version 5.0.^30^ The long-term AE groups were categorized as follows:

- Late cytopenias: grade 3 or higher cytopenias, or those that require treatment with granulocyte colony-stimulating factor, thrombopoietin receptor agonists, and/or transfusions.
- Hypogammaglobulinemia: patients with immunoglobulin G (IgG) levels less than 400 mg/dL (to convert to grams per liter, multiply by 0.01) and/or serious or recurrent infections who received intravenous immunoglobulin replacement therapy.^12,31^
- Infections: any infection documented in the medical records.
- Subsequent neoplasms: any malignant neoplasm with pathologic confirmation, different from the original lymphoma diagnosis.
- Dermatologic diseases: any new or persisting skin lesion.
- Immune-related events: any immune-mediated disorder.
- Psychiatric and neurologic disorders.
- Cardiovascular events: clinical, rhythm- or contractility-related, or other echocardiographic or electrocardiographic disturbances.
- Graft-vs-host disease: for those with a previous allogeneic hematopoietic cell transplant, only events that require specific treatment.
- Other events: any AEs not described in previous categories and that met the time criteria.

### AE Episodes

Episodes were created for every AE. If a certain AE occurred after complete resolution of the previous event for a particular patient, it was considered a new AE; therefore, 1 patient could have more than 1 episode per each AE. Start and end dates of AEs were reviewed at every follow-up study visit. When an AE end date was not reported, the AE episode remained open. In addition, to confirm the evolution of each AE episode, information on the status of the events (unresolved, resolved, resolved with sequels) was reviewed at every follow-up visit. If the AE did not have a reported end date during our study period, the end date of the study or the date of patient withdrawal was used to calculate the length of the AE episodes.

### Statistical Analysis

Baseline patient demographic and clinical characteristics were described as mean (SD) values or median (IQR) values for continuous variables and as frequencies and percentages for categorical variables. The χ^2^ test was used to compare categorical variables, and the Mann-Whitney test was used for continuous variables, to make comparisons between the CAR T-cell products.

The incidence rate of each AE was calculated per 100 patient-months. Poisson regression analysis was used to explore the difference in the incidence between the CAR T-cell products. Confidence limits and significance tests were corrected after checking for overdispersion.^32^ When the number of episodes was zero, exact Poisson confidence limits were computed.

In addition, latencies and the length of AE episodes were calculated and reported as median values, quartiles, and minimum and maximum values. Cumulative incidence at 12 and 24 months was used to estimate NRM, defined as death after CAR T-cell infusion without relapse or progression to lymphoma disease. The data analyses were carried out using SAS, version 9.4 (SAS Institute Inc). All *P* values were from 2-sided tests, and results were deemed statistically significant at *P* < .05.

## Results

### Patients

During the study period, 391 patients with relapsed or refractory LBCL received commercial CAR T-cell therapy in the 6 participating centers. Of these 391 patients, 172 (44.0%; mean [SD] age, 58.5 [13.7] years; 101 men [58.7%] and 71 women [41.3%]) were alive at 3 months after CAR T-cell infusion without subsequent lymphoma-directed therapy and were enrolled in the study.

Diffuse LBCL was the most common diagnosis (126 [73.3%]), with a median of 2 (IQR, 2-3) prior lines of therapy (Table 1). Regarding the CAR T-cell construct, 117 (68.0%) received axicabtagene ciloleucel, and 55 (32.0%) received tisagenlecleucel. Patients treated with tisagenlecleucel were older (median age, 66.0 years [IQR, 56.0-72.0 years] vs 60.0 years [IQR, 47.0-68.0 years]; *P* = .01) and had a lower rate of primary refractory disease (12.7% [7 of 55] vs 29.9% [35 of 117]; *P* = .047), in comparison with the axicabtagene ciloleucel subgroup. Additional demographic and clinical characteristics were balanced between both subgroups and are summarized in Table 1.

**Table 1.** Baseline Characteristics of Patients Receiving CAR T-Cell Infusions

Characteristic	Total (N = 172)	CAR T-cell product		*P* value
		Tisagenlecleucel (n = 55 [32.0%])	Axicabtagene ciloleucel (n = 117 [68.0%])	
Sex, No. (%)				
Female	71 (41.3)	22 (40.0)	49 (41.9)	.81
Male	101 (58.7)	33 (60.0)	68 (58.1)	
Age, y				
Mean (SD)	58.5 (13.7)	62.3 (13.6)	56.7 (13.5)	.01
Median (IQR)	61.0 (50.0-69.0)	66.0 (56.0-72.0)	60.0 (47.0-68.0)	
CAR T-cell indication, No. (%)				
Diffuse large B-cell lymphoma	126 (73.3)	44 (80.0)	82 (70.1)	.07
Transformed from follicular lymphoma	23 (13.4)	9 (16.4)	14 (12.0)	
T-cell or histiocyte-rich large B-cell lymphoma	3 (1.7)	0	3 (2.6)	
Primary mediastinal lymphoma	20 (11.6)	2 (3.6)	18 (15.4)	
No. of previous lines of therapy, median (IQR)	2 (2-3)	3 (2-3)	2 (2-3)	.28
Previous SCT, No. (%)				
Autologous	65 (37.8)	25 (45.5)	40 (34.2)	.29
Allogeneic	3 (1.7)	1 (1.8)	2 (1.7)	
None	104 (60.5)	29 (52.7)	75 (64.1)	
Response to previous therapy, No. (%)				
Primary refractory	42 (24.6)	7 (12.7)	35 (29.9)	.047
Refractory to last therapy	56 (32.7)	22 (40.7)	34 (29.1)	
Relapsed	73 (42.7)	25 (46.3)	48 (41.0)	

Abbreviations: CAR, chimeric antigen receptor; SCT, stem cell transplant.

The median follow-up for all enrolled patients was 13.9 months (IQR, 8.2-23.8 months). At the 3-month postinfusion time point (study inclusion), 132 patients (76.7%) were in complete remission, 31 patients (18.0%) in partial remission, and 9 patients (5.2%) in stable disease. At the end of the study, 119 patients (69.2%) remained in complete remission, 6 (3.5%) were in partial remission, 14 (8.1%) in stable disease, and 33 (19.2%) had experienced disease progression. Reasons for study discontinuation included new antilymphoma therapy (n = 33), lost to follow-up (n = 4), and death (n = 7).

### Long-Term AE Overview

A total of 442 long-term AEs of any grade were registered for 135 (78.5%) patients. Grade 3 to 4 AEs were noted for 75 patients (43.6%), and 7 patients (4.1%) experienced fatal AEs. The most-frequent long-term AEs were infections (146 events in 79 of 172 patients [45.9%]) and neutropenia (93 events in 69 of 172 patients [40.1%]) (eTable 1 in Supplement 1). Taking the CAR T-cell construct into consideration, the incidence of all registered AEs was similar for patients receiving axicabtagene ciloleucel and those receiving tisagenlecleucel, except for a higher incidence of thrombocytopenia among patients receiving tisagenlecleucel (eTables 2 and 3 in Supplement 1).

### Infections and Hypogammaglobulinemia

A total of 146 infectious events were reported for 79 of 172 patients (45.9%), with an incidence of 5.6 per 100 person-months (95% CI, 4.5-7.0 per 100 person-months) (Figure; eTable 1 in Supplement 1). A total of 93 of 172 patients (54.1%) did not experience any infectious event during follow-up.

**Figure.**
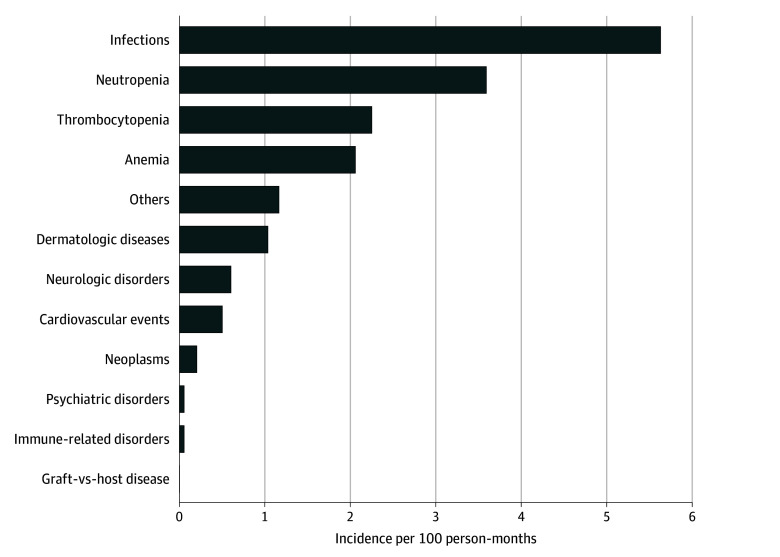
Long-Term Adverse Event Incidence Per 100 Person-Months

Of the 146 total episodes, only 18 (12.3%) had started within 3 months after infusion and persisted at the time of patient inclusion (eTable 4 in Supplement 1). The remaining events (127 [87.0%]) started beyond the 3-month landmark, with a median onset at 271 days (range, 98-751 days) after infusion. The number of infectious episodes remained stable across time during the study period. During the 18- to 24-month period, of 14 patients who experienced an infectious event, for 8 patients (57.1%) it was their first infectious event, for 5 (35.7%) it was the second infectious event, and only in 1 patient (7.1%) was it the third infectious episode.

The most common type of infection was COVID-19 (44 patients [25.6%]; 45 episodes). The median latency period was 307 days (range, 39-751 days), and the median length was 37 days (range, 3-152 days). Other frequent infectious events included acute lower respiratory tract infections of unspecified cause, acute upper respiratory tract infections, and cystitis (see eTable 7 Supplement 1 for all infectious episodes). After SARS-CoV-2, the most common microbiological isolates were *Clostridium difficile*, *Pseudomonas aeruginosa*, *Campylobacter*, herpes zoster, influenza virus, and respiratory syncytial virus.

Considering severity, 55 grade 3 or higher infectious events were documented, including COVID-19 infections (n = 20), pneumonia without microbiological isolation (n = 4), unspecified acute upper respiratory tract infections (n = 4), pneumonia due to *Pseudomonas* (n = 3), and herpes zoster (n = 2). Regarding the hypogammaglobulinemia variable (as defined in Methods), 78 patients (45.3%) had IgG levels less than 400 mg/dL; of those, 33 (19.2%) received immunoglobulin replacement. In addition, 10 patients (5.8%) received immunoglobulin replacement because of serious or repeated infections and an IgG level of 400 mg/dL or more. After 24 months of follow-up, 17 patients had ongoing hypogammaglobulinemia.

### Hematologic Toxic Effects

Neutropenia of any grade was the second most frequent AE (69 patients [40.1%]; 93 episodes) with an incidence of 3.6 per 100 person-months (95% CI, 2.9-4.5 per 100 person-months) (Figure; eTable 1 in Supplement 1). Thirty-five episodes (37.6%) started within 3 months after infusion and were persistent after this time point, while 58 episodes (62.4%) had a new onset after the 3-month landmark for patients with previous neutrophil recovery (eTable 4 in Supplement 1). The frequency of neutropenia decreased after 6 months after the infusion (eTables 5 and 6 in Supplement 1). The median latency period from CAR T-cell infusion for the total number of neutropenia episodes of the study was 111 days (IQR, 57-211 days), and the median length for the 53 episodes (57.0%) with a known end AE date was 92 days (IQR, 28-185 days) (Table 2).

**Table 2.** Latency and Length of Adverse Event Episodes

Adverse event	Latency		Length^a^	
	Episodes, No.	Median (IQR) [range], d	Episodes with confirmed end date, No.	Median (IQR) [range], d
Infections	145^b^	251 (158-400) [9-751]	120	15 (9-34) [2-173]
Neutropenia	93	111 (57-211) [0-692]	53	92 (28-185) [5-495]
Thrombocytopenia	58	70 (1-258) [0-558]	19	126 (60-189) [9-556]
Anemia	53	90 (6-186) [0-721]	19	96 (35-148) [1-533]
Others	29^b^	191 (84-363) [2-754]	6	60 (3-97) [1-224]
Dermatologic diseases	27	165 (106-282) [57-518]	8	127 (70-152) [25-212]
Neurologic disorders	15	171 (63-279) [8-487]	10	169 (83-270) [1-295]
Cardiovascular events	13	265 (155-305) [0-752]	7	6 (1-126) [1-308]
Secondary neoplasms	5	514 (374-541) [217-693]	3	104 (29-433) [29-433]
Psychiatric disorders	1	550 (550-550) [550-550]	0	0
Immune-related events	1	458 (458-458) [458-458]	0	NA
Graft-vs-host disease	0	NA	0	NA

Abbreviation: NA, not applicable.

^a^ Episodes without confirmed end date are not included.

^b^ One episode with missing start date.

In terms of severity, grade 3 or greater neutropenia was observed in 49 patients (28.5%; 63 episodes), with a median latency period after CAR T-cell infusion of 99 days (range, 5-495 days). Most of these episodes (38 [60.3%]) had an onset later than 3 months after infusion, with a median latency period in this subgroup of 185 days (range, 91-632 days) after infusion (eTables 3 and 6 in Supplement 1).

Thrombocytopenia of any grade was observed in 46 patients (26.7; 58 episodes) and was the third most frequent AE, with an incidence of 2.2 per 100 person-months (95% CI, 1.7-3.0 per 100 person-months) (eTable 1 in Supplement 1). Most episodes started within 3 months after infusion and lasted beyond that time point (eTable 4 in Supplement 1). Again, the frequency decreased after 6 months after infusion (eTable 5 in Supplement 1). The median latency period was 70 days (IQR, 1-258 days) (Table 2), and grade 3 or higher events were reported for 22 patients (12.8%; 25 episodes).

Regarding anemia, 44 patients (25.6%) with 53 episodes were reported, of which 14 patients (8.1%; 14 episodes) had episodes that were grade 3 or higher. Most episodes (28 of 53 [52.8%]) had started during the first 3 months after infusion (eTable 4 in Supplement 1).

### Dermatologic Diseases

Twenty-seven cutaneous AEs were reported for 23 patients (13.4%). Eczematous dermatoses and eosinophilic dermatoses were the most frequent (5 each [2.9%]). Additional skin events are described in eTable 8 in Supplement 1. Most (88.9% [24 of 27]) dermatologic AEs started beyond 3 months after infusion. There were no reported grade 3 or higher dermatologic events. Five cases were unresolved at the end of follow-up.

### Neurologic Events

Fifteen patients (8.7%) had neurologic AEs (eTable 1 in Supplement 1), with 10 of the 15 episodes (66.7%) developing later than 3 months after CAR T-cell infusion (eTable 4 in Supplement 1). The most frequent events were paresthesias in 4 patients (2.3%), followed by unspecified encephalopathy (3 [1.7%]) (eTable 9 in Supplement 1). Other neurologic AEs had only 1 case each. Regarding severity, nontraumatic subdural hemorrhage was the only grade 4 AE. All cases of encephalopathy were grade 3, and the other 11 neurological AEs were all grade 2 or less. Four neurologic events were unresolved at the end of the study follow-up (paresthesias, central vertigo, and 2 patients with encephalopathy).

### Cardiovascular Events

Ten patients (5.8%) had 13 cardiovascular AEs (eTable 1 in Supplement 1), with heart failure (6 [3.5%]) being the most frequent (eTable 10 in Supplement 1). Three patients had more than 1 cardiovascular AE: the first patient (with a history of reduced left ventricular ejection fraction previous to CAR T-cell therapy) presented with syncope at 9 months and worsening heart failure at 24 months after the infusion, the second patient (without prior cardiovascular disease) developed an acute episode of pericarditis at 3 months and thoracic pain at 12 months after the infusion, and the third patient developed 2 episodes of worsening heart failure at 3 and 12 months after CAR T-cell therapy. Only one cardiovascular event was grade 3: a 75-year-old woman with acute heart failure and de novo reduced left ventricular ejection fraction (41%) at 6 months after infusion, requiring hospital admission and intensive diuretic treatment; this patient had a concomitant severe COVID-19 infection. Two other patients with concomitant COVID-19 infection presented with 2 cardiovascular events without clinical repercussion (atrial fibrillation and hypertensive cardiomyopathy). There was only one unresolved event at end of study follow-up: an incipient interstitial fibrosis that did not require treatment.

### Secondary Malignant Neoplasms

There were 5 cases of secondary malignant neoplasms diagnosed in 4 (2.3%) patients, with an incidence of 0.2 per 100 person-months (95% CI, 0.1-0.4 per 100 person-months) (eTable 1 in Supplement 1). Two patients developed skin malignant neoplasms, which were surgically removed (1 patient with melanoma and a subsequent nonmelanoma skin cancer and 1 patient with a basal cell carcinoma) (Table 3).^30^ A female patient developed infiltrating ductal breast carcinoma, which required tumor resection and radiotherapy. Finally, a male patient received a diagnosis of therapy-related myeloid neoplasm (myelodysplastic syndrome) at 23 months after CAR T-cell infusion. The median latency period for these second neoplasms was 514 days (IQR, 374-541 days) (Table 2). No cases of T-cell malignant neoplasms were reported during the study period with a median follow-up of 13.9 months (IQR 8.2-23.8 months).

**Table 3.** Secondary Malignant Neoplasms

Sex/age, y	CAR T-cell product	Diagnosis	Severity (CTCAE grade)	Time to onset, d	Treatment	Final status at withdrawal or end of study
Female/60s	Axicabtagene ciloleucel	Infiltrating ductal breast carcinoma	3	217	Tumor resection and radiotherapy	Recovered
Male/70s	Axicabtagene ciloleucel	Melanoma skin cancer	2	374	Surgical excision	Recovered
		Cutaneous squamous cell carcinoma	2	514	Surgical excision	Recovered
Male/70s	Axicabtagene ciloleucel	Basal cell carcinoma	3	541	Surgical excision	Recovered
Male/70s	Axicabtagene ciloleucel	Therapy-related myelodysplastic syndrome, *TP53* variant and abnormalities of chromosomes 5 and 7	4	693	Azacitidine	Unresolved

Abbreviation: CTCAE, Common Terminology Criteria for Adverse Events, version 5.0.^30^

### Other AEs

In our study, 25 patients (14.5%) had 30 AEs that were classified as “other.” These included diarrhea (n = 4), abnormal laboratory test results (n = 3), dyslipidemia (n = 2), and embolism and thrombosis (n = 2) as the most frequent (eTable 11 in Supplement 1). The median latency period was 191 days (IQR, 84-363 days), with most of them (n = 21) starting after 3 months after infusion. Only 4 events were grade 3: a pulmonary embolism, interstitial pulmonary disease, idiopathic thrombocytopenic purpura, and idiopathic hepatitis. Nine patients had unresolved AEs at the end of the follow-up.

The only immune-related AE was described for a 69-year-old male patient, who developed nodular sarcoidosis at 18 months after tisagenlecleucel infusion, without requiring therapy. No cases of graft-vs-host disease were observed among the 3 patients with a previous allogeneic hematopoietic cell transplant.

### Nonrelapse Mortality

Seven patients (4.1%) experienced NRM during study follow-up, all from infections: 3 patients from COVID-19 pneumonia, 1 patient with *Escherichia coli* and *Achromobacter* sepsis, 1 with *P aeruginosa* sepsis, 1 with *P aeruginosa* and *E coli* pneumonia, and 1 with *Streptococcus pneumoniae* pneumonia. The latter 2 patients had a concomitant persistent COVID-19 infection. Two of the 7 patients who died had IgG levels lower than 400 mg/dL, and 1 was receiving intravenous immunoglobulin replacement. The cumulative incidence of NRM was 4.4% at 12 months and 6.3% at 24 months (eFigure in Supplement 1).

## Discussion

In this prospective, multicenter, observational cohort study, we comprehensively addressed late AEs occurring after CAR T-cell therapy among patients with aggressive B-cell lymphoma who were alive at 3 months after infusion, without subsequent antilymphoma therapy. Infections and hematologic toxic effects were the most common events reported for these patients beyond the 3-month landmark. We identified skin, neurologic, and cardiac AEs at an intermediate frequency. In addition, secondary malignant neoplasms were diagnosed in 2.3% of patients and no cases of T-cell lymphoma were identified in our series. These AEs were associated with late NRM events in 7 patients (4.1%), all of them from infection.

In our study, infections were the most common late AE after therapy, as seen in previous retrospective studies.^31,33,34,35^ Respiratory tract infections were the most common, while COVID-19 was the most frequent isolate. The prevalence of COVID-19 infections among patients receiving CAR T-cell therapy is not well known, as most studies have focused exclusively on the outcome of patients who had already received a diagnosis of this infection or the studies were not designed to capture such incidence. Busca et al^36^ retrospectively studied 459 consecutive patients receiving CAR T-cell therapy with lymphoma and multiple myeloma and reported a prevalence of 4.8% in approximately 1 year of follow-up. This and other studies^37^ reported a high mortality rate (41.1%) of COVID-19 infection in this patient population. Here, we report an increased incidence of COVID-19 infection (25.6%) and lower mortality (3 of 44 [6.8%]) compared with the other studies, probably because our prospective design allowed us to capture better the real incidence of this infection, including mild cases that were overlooked in other ad hoc studies and/or because patients were vaccinated, although we did not collect information on COVID-19 vaccination. Other frequent late infectious events included respiratory tract infections, followed by urinary tract and gastrointestinal infections. A microbiological isolate could not be detected in most of these events. Among those with microbiological identification, respiratory viruses and herpes zoster were the most frequently identified.

Hematologic toxic effects were the second most common AEs, and neutropenia was the most frequent cytopenia. These findings are in accordance with the data provided by other studies, where prolonged cytopenias were described as one of the most predominant complications after CAR T-cell therapy.^11^ Even though some patients with hematologic toxic effects had an early onset (before the 3-month landmark), around two-thirds experienced a cytopenia after full recovery of their blood cell counts. This finding is in line with the bimodal cytopenia patterns described in previous publications.^11^ Identifying the cause of these late cytopenias is beyond the scope of our study and should be addressed in future analyses; plausible causes include poor bone marrow reserve, concurrent infections, and/or undiagnosed myelodysplastic syndrome.

Dermatologic events after CAR T-cell therapy are not well characterized. We identified 27 of these events in 23 patients (13.4%). In fact, we include here to our knowledge the largest series of patients with skin toxic effects after CAR T-cell therapy, as most of these types of events have been described in case reports or small series.^38,39^ Unlike other studies in which dermatologic AEs were grouped in the immune-related section,^12^ we were able to characterize them in detail. In our study, most of the cutaneous AEs were mild and included a wide variety of clinical presentations. In addition, 3 skin cancers were reported in 2 patients but were categorized as secondary malignant neoplasms. Concerning the time of presentation, some dermatologic events occurred more than 1 year after CAR T-cell infusion. This finding highlights the need for continuous surveillance of skin lesions and dermatologic consultations for these patients.

Cardiovascular and neurologic AEs were described in less than 10% of patients, with the most frequent cardiovascular AE being heart failure and the most frequent neurologic AE being paresthesias. However, most of these events were mild and resolved after pharmacologic therapy. Worsening heart failure was the leading cardiovascular event, despite a normal ejection fraction being required per protocol before CAR T-cell therapy. Regarding neurovascular AEs, 2 cerebral hemorrhages were identified in this series but, as in the ZUMA-1 trial, these events were infrequent.^1^

Secondary malignant neoplasms are currently in the spotlight after the FDA announced a boxed warning for BCMA (B-cell maturation antigen)–directed and CD19-directed autologous CAR T-cell therapy.^18^ This announcement was based on 22 cases of secondary malignant neoplasms diagnosed, with a latency period ranging from 1 to 19 months (50% occurring in the first year after infusion).^40,41,42,43,44^ In our study, with prospective epidemiologic surveillance covering the incidence period reported by the FDA, we did not identify any cases of T-cell malignant neoplasms. Regarding other secondary malignant neoplasms, and in line with our findings, myeloid neoplasms and skin cancers are the most frequently reported after CAR T-cell therapy,^45^ both in clinical trials^12^ and clinical practice studies.^46,47^ However, the frequency of myeloid neoplasms is low compared with other studies.^45,46,47^ This low frequency may be due to the end of follow-up if they started a new treatment for lymphoma, or at 2 years after patient infusion, whichever came first. Also, as patients were followed up at their local referral sites, some cases could have been missed.

To our knowledge, this is one of the first prospective studies analyzing late NRM in patients receving CAR T-cell treatment. Infection was the main cause of NRM in our patient population, as in previous studies.^31,48^ In the current study, cumulative incidence of NRM among patients surviving 3 months without further lymphoma-directed therapy at 12 months was 4.4%. We consider this information valuable for patient counselling before CAR T-cell treatment and in the 3-month visit, when most patients return to their referring center.

### Limitations

Our research has some limitations. First, the study protocol had a preestablished maximum patient follow-up of 24 months, potentially underrepresenting late AEs with a longer latency period, such as secondary malignant neoplasms. Second, despite the relatively large sample size of the study (taking into account that only patients not receiving additional therapy after 3 months after the infusion were included), we had limited power to detect very infrequent events harboring an incidence of less than 1%. In addition, the results of this study can be applied only to patients who have not experienced progressive disease by the 3-month postinfusion time point. However, we consider that some of these limitations are counterbalanced by the prospective and multicenter design of the study.

## Conclusions

In this cohort study on long-term safety of CAR T-cell therapy, late AEs were frequent after CAR T-cell therapy, with infections and cytopenias being the most common events. Despite most of the AEs being manageable, some patients experienced severe toxic effects long after infusion. A comprehensive approach to these long-term events is warranted to ensure optimal diagnosis and early intervention in CAR T-cell recipients.

## Notes

Supplement 1. **eTable 1.** Long-Term AEs Incidence per 100 Person-Months (PM) **eTable 2.** Long-Term AE Incidence Rate per 100 Person-Months (PM) per Infused CAR T-Cell Product **eTable 3.** Grade ≥3 Infectious and Cytopenia Incidence Rate Per 100 Person-Months (PM) Per Infused CAR T-Cell Product **eTable 4.** Latency and Length of AE Episodes, According to Time of Onset **eTable 5.** Any Grade AE Episodes According to Onset **eTable 6.** Grade ≥3 AE Episodes According to Onset **eTable 7.** All Infectious Episodes **eTable 8.** Dermatologic Adverse Events **eTable 9.** Neurologic Adverse Events **eTable 10.** Cardiovascular Adverse Events **eTable 11.** Other Adverse Events **eFigure.** Cumulative Incidence of NRM

Supplement 2.
Data Sharing Statement
