# Supplementary material for: Late Adverse Events After Chimeric Antigen Receptor T-Cell Therapy for Patients With Aggressive B-Cell Non-Hodgkin Lymphoma
Source: JAMA Netw Open. 2025 Feb 25;8(2):e2461683. doi: 10.1001/jamanetworkopen.2024.61683 (PMC11862965; doi:10.1001/jamanetworkopen.2024.61683)
Supplement: Supplement 1. — eTable 1. Long-Term AEs Incidence per 100 Person-Months (PM) eTable 2. Long-Term AE Incidence Rate per 100 Person-Months (PM) per Infused CAR T-Cell Product eTable 3. Grade ≥3 Infectious and Cytopenia Incidence Rate Per 100 Person-Months (PM) Per Infused CAR T-Cell Product eTable 4. Latency and Length of AE Episodes, According to Time of Onset eTable 5. Any Grade AE Episodes According to Onset eTable 6. Grade ≥3 AE Episodes According to Onset eTable 7. All Infectious Episodes eTable 8. Dermatologic Adverse Events eTable 9. Neurologic Adverse Events eTable 10. Cardiovascular Adverse Events eTable 11. Other Adverse Events eFigure. Cumulative Incidence of NRM [file jamanetwopen-e2461683-s001.pdf]

Supplementary Online Content

Camacho-Arteaga L, Iacoboni G, Kwon M, et al. Late adverse events after chimeric antigen receptor T-cell therapy for patients with aggressive B-cell non-Hodgkin lymphoma. *JAMA Netw Open.* 2025;8(2):e2461683.
doi:10.1001/jamanetworkopen.2024.61683

eTable 1. Long-Term AEs Incidence per 100 Person-Months (PM)

eTable 2. Long-Term AE Incidence Rate per 100 Person-Months (PM) Per Infused CAR T-Cell Product

eTable 3. Grade ≥3 Infectious and Cytopenia Incidence Rate Per 100 Person-Months (PM) Per Infused CAR T-Cell Product

eTable 4. Latency and Length of AE Episodes, According to Time of Onset

eTable 5. Any Grade AE Episodes According to Onset

eTable 6. Grade ≥3 AE Episodes According to Onset

eTable 7. All Infectious Episodes

eTable 8. Dermatologic Adverse Events

eTable 9. Neurologic Adverse Events

eTable 10. Cardiovascular Adverse Events

eTable 11. Other Adverse Events

eFigure. Cumulative Incidence of NRM

This supplementary material has been provided by the authors to give readers additional information about their work.

© 2025 Camacho-Arteaga L et al. *JAMA Network Open.*

eTable 1. Long-Term AEs Incidence Per 100 Person-Months (PM)

Adverse Events	Patients No. (%)	Episodes	Incidence rate per 100PM	95% CI
Infections	79 (45.9)	146	5.63	[4.50-7.04]
Neutropenia	69 (40.1)	93	3.59	[2.89-4.53]
Thrombocytopenia	46 (26.7)	58	2.24	[1.65-3.02]
Anemia	44 (25.6)	53	2.04	[1.48-2.83]
Others	25 (14.5)	30	1.16	[0.74-1.82]
Dermatological diseases	23 (13.4)	27	1.04	[0.71-1.54]
Neurologic disorders	15 (8.7)	15	0.58	[0.34-0.99]
Cardiovascular events	10 (5.8)	13	0.50	[0.26-0.97]
Secondary neoplasms	4 (2.3)	5	0.19	[0.09-0.44]
Psychiatric disorders	1 (0.6)	1	0.04	[0.01-0.18]
Immune-related events	1 (0.6)	1	0.04	[0.01-0.23]
GVHD	0 (0)	0	0	[0-0.14]

© 2025 Camacho-Arteaga L et al. *JAMA Network Open.*

eTable 2. Long-Term AE Incidence Rate Per 100 Person-Months (PM) Per Infused CAR T-Cell Product

	Tisagenlecleucel (n=55)				***Axicabtagen ciloleucel (n=117)***				
Adverse Events	***Patients n (%)***	***Episodes***	***Incidence rate per 100PM***	***95% CI***	***Patients n (%)***	***Episodes***	***Incidence rate per 100PM***	***95% CI***	***p-value***
Infections	19 (34.5)	31	4.10	[2.42-6.94]	60 (51.3)	115	6.26	[4.88-8.02]	0.133
Neutropenia	23 (41.8)	33	4.37	[2.83-6.75]	46 (39.3)	60	3.27	[2.49-4.28]	0.233
Thrombocytopenia	19 (34.5)	26	3.44	[2.14-5.54]	27 (23.1)	32	1.74	[1.20-2.53]	0.022
Anemia	16 (29.1)	20	2.65	[1.50-4.66]	28 (23.9)	33	1.80	[1.21-2.66]	0.249
Others	10 (18.2)	13	1.72	[0.80-3.69]	15 (12.8)	17	0.93	[0.54-1.57]	0.161
Dermatological diseases	6 (10.9)	7	0.93	[0.37-2.35]	17 (14.5)	20	1.09	[0.72-1.65]	0.724
Neurologic disorders	7 (12.7)	7	0.93	[0.40-2.12]	8 (6.8)	8	0.44	[0.22-0.88]	0.159
Cardiovascular events	5 (9.1)	7	0.93	[0.37-2.30]	5 (4.3)	6	0.33	[0.13-0.82]	0.108
Neoplasms	0 (0)	0	0	[0-0.49]	4 (3.4)	5	0.27	[0.12-0.62]	0.960
Psychiatric disorders	0 (0)	0	0	[0-0.49]	1 (0.9)	1	0.05	[0.01-0.27]	0.960
Immune-related events	1 (1.8)	1	0.13	[0.02-0.76]	0 (0)	0	0	[0-0.20]	0.943
GVHD	0 (0)	0	0	[0-0.49]	0 (0)	0	0	[0-0.20]	-

© 2025 Camacho-Arteaga L et al. *JAMA Network Open.*

eTable 3. Grade ≥3 Infectious and Cytopenia Incidence Rate Per 100 Person-Months (PM) Per Infused CAR T-Cell Product

	Tisagenlecleucel (n=55)				***Axicabtagen ciloleucel (n=117)***				
Adverse Events	***Patients n (%)***	***Episodes***	***Incidence rate per 100PM***	***95% CI***	***Patients n (%)***	***Episodes***	***Incidence rate per 100PM***	***95% CI***	***p-value***
Infections	*10 (18.2)*	*15*	*1.98*	*[0.87-4.53]*	*25 (21.4)*	*40*	*2.18*	*[1.44-3.28]*	*0.829*
Neutropenia	*15 (27.3)*	*24*	*3.18*	*[1.82-5.53]*	*34 (29.1)*	*39*	*2.12*	*[1.50-3.01]*	*0.193*
Thrombocytopenia	*8 (14.5)*	*10*	*1.32*	*[0.57-3.09]*	*14 (12.0)*	*15*	*0.82*	*[0.45-1.48]*	*0.333*
Anemia	*6 (10.9)*	*6*	*0.79*	*[0.29-2.18]*	*8 (6.8)*	*8*	*0.44*	*[0.17-1.11]*	*0.396*

© 2025 Camacho-Arteaga L et al. *JAMA Network Open.*

eTable 4. Latency and Length of AE Episodes, According to Time of Onset

	Onset within 3 months and persisting beyond						***Onset after 3 months***					
	Episodes	***Latency (median [IQR] Min-max)***	***Episodes with confirmed end date***	***Length (median [IQR] Min-max)***	***Episodes w/out confirmed end date***	***Length (median [IQR] Min-max)***	***Episodes***	***Latency (median [IQR] Min-max)***	***Episodes with confirmed end date***	***Length (median [IQR] Min-max)***	***Episodes w/out confirmed end date***	***Length (median [IQR] Min-max)***
Infections	18	64.5 [42-84] 9-91	12	30.5 [17.5-114.5] 7-173	6	337 [277-494] 116-664	127*	271 [184-421] 98-751	108	14 [8-32.5] 2-165	19	202 [35-258] 3-558
Neutropenia	35	29 [1-64] 0-91	18	184 [102-287] 12-495	17	375 [150-494] 23-728	58	184 [123-336] 90-692	35	64 [21-100] 5-478	23	164 [42-394] 2-616
Thrombocytopenia	31	1 [0-30] 0-86	9	162 [126-189] 9-556	22	372 [251-572] 44-803	27	284 [151-365] 91-558	10	86 [32-152] 15-215	17	291 [240-356] 1-674
Anemia	28	11 [0-60.5] 0-91	11	140 [107-196] 21-533	17	329 [263-572] 44-728	25	259 [158-380] 91-721	8	50.5 [7-91.5] 1-96	17	84 [19-270] 0-357
Others	8	33.5 [14.5-55.5] 2-84	3	97 [68-224] 68-224	5	391 [262-462] 104-728	21*	278 [166-525] 95-754	3	3 [1-52] 1-52	18	145 [46-301] 19-542
Dermatological diseases	3	61 [57-78] 57-78	2	193.5 [175-212] 175-212	1	262 [262] 262	24	177 [119-289] 92-518	6	115 [35-128] 25-129	18	307.5 [203-595] 35-628
Neurologic disorders	5	63 [60-63] 8-67	4	167.5 [109.5-246.5]	1	82 [82] 82	10	266.5 [171-334]	6	185.5 [3-270] 1-295	4	368 [165.5-516]

© 2025 Camacho-Arteaga L et al. *JAMA Network Open.*

				83-294				131-487				54-573
Cardiovascular events	1	0<br>[0]<br>0	0	-	1	409<br>[409]<br>409	12	268<br>[164.5-319]<br>117-752	7	6<br>[1-126]<br>1-308	5	138<br>[87-287]<br>35-645
Neoplasms	0	-	0	-	0	-	5	514<br>[374-541]<br>217-693	3	104<br>[29-433]<br>29-433	2	127.5<br>[40-215]<br>40-215
Psychiatric disorders	0	-	0	-	0	-	1	550<br>[550]<br>550	0	-	1	189<br>[189]<br>189
Immune-related events	0	-	0	-	0	-	1	458<br>[458]<br>458	0	-	1	87<br>[87]<br>87
GVHD	0	-	0	-	0	-	0	-	0	-	0	-

*One episode with missing start date

© 2025 Camacho-Arteaga L et al. *JAMA Network Open.*

eTable 5. Any Grade AE Episodes According to Onset*

Adverse Events	***3 months (n=172) n (%)***		***>3 - 6 months (n=171) n (%)***		***>6 - 9 months (n=148) n (%)***		***>9 - 12 months (n=124) n (%)***		***>12 - 18 months (n=102) n (%)***		***>18 - 24 months (n=68) n (%)***	
Patients with any AE	73 (42.4)		64 (37.4)		43 (29.1)		37 (29.8)		35 (34.3)		22 (32.4)	
	Patients	***Episodes***	***Patients***	***Episodes***	***Patients***	***Episodes***	***Patients***	***Episodes***	***Patients***	***Episodes***	***Patients***	***Episodes***
Infections	18 (10.5)	18	26 (15.2)	30	27 (18.2)	36	15 (12.1)	18	17 (16.7)	21	14 (20.6)	23
Neutropenia	34 (19.8)	35	25 (14.6)	27	10 (6.8)	10	9 (7.3)	10	7 (6.9)	7	4 (5.9)	4
Thrombocytopenia	31 (18)	31	8 (4.7)	9	3 (2.0)	4	7 (5.6)	7	6 (5.9)	6	1 (1.5)	1
Anemia	28 (16.3)	28	7 (4.1)	9	4 (2.7)	4	5 (4.0)	5	5 (4.9)	5	2 (2.9)	2
Others	6 (3.5)	8	6 (3.5)	6	5 (3.4)	5	4 (3.2)	4	2 (2.0)	2	5 (7.4)	5
Dermatological diseases	3 (1.7)	3	11 (6.4)	12	5 (3.4)	5	4 (3.2)	4	3 (2.9)	3	0 (0)	0
Neurologic disorders	5 (2.9)	5	4 (2.3)	4	1 (0.7)	1	3 (2.4)	3	2 (2.0)	2	0 (0)	0
Cardiovascular events	1 (0.6)	1	4 (2.3)	4	3 (2.0)	3	3 (2.4)	3	0 (0)	0	2 (2.9)	2
Neoplasms	0 (0)	0	0 (0)	0	1 (0.7)	1	0 (0)	0	2 (2.0)	3	1 (1.5)	1
Psychiatric disorders	0 (0)	0	0 (0)	0	0 (0)	0	0 (0)	0	0 (0)	0	1 (1.5)	1
Immune-related events	0 (0)	0	0 (0)	0	0 (0)	0	0 (0)	0	1 (1.0)	1	0 (0)	0
GVHD	0 (0)	0	0 (0)	0	0 (0)	0	0 (0)	0	0 (0)	0	0 (0)	0

*One patient could have more that one AE episode

© 2025 Camacho-Arteaga L et al. *JAMA Network Open.*

eTable 6. Grade ≥3 AE Episodes According to Onset

Adverse Events	3 months (n=172) n (%)		>3 - 6 months (n=171) n (%)		>6 - 9 months (n=148) n (%)		>9 - 12 months (n=124) n (%)		>12 - 18 months (n=102) n (%)		>18 - 24 months (n=68) n (%)	
Patients with any grade 3-5 AE	42 (24.4)		25 (14.6)		20 (13.5)		11 (8.9)		12 (11.8)		9 (13.2)	
	**Patients**	**Episodes**	**Patients**	**Episodes**	**Patients**	**Episodes**	**Patients**	**Episodes**	**Patients**	**Episodes**	**Patients**	**Episodes**
Infections	8 (4.7)	8	10 (5.8)	10	14 (9.5)	15	6 (4.8)	6	4 (3.9)	4	6 (8.8)	12
Neutropenia	25 (14.5)	26	15 (8.8)	17	7 (4.7)	7	5 (4.0)	6	5 (4.9)	5	2 (2.9)	2
Thrombocytopenia	15 (8.7)	15	4 (2.3)	5	1 (0.7)	1	3 (2.4)	3	1 (1.0)	1	0 (0)	0
Anemia	7 (4.1)	7	3 (1.8)	3	2 (1.4)	2	0 (0)	0	2 (2.0)	2	0 (0)	0
Others	1 (0.6)	1	0 (0)	0	0 (0)	0	1 (0.8)	1	0 (0)	0	2 (2.9)	2
Dermatological diseases	0 (0)	0	0 (0)	0	0 (0)	0	0 (0)	0	0 (0)	0	0 (0)	0
Neurologic disorders	2 (1.2)	2	2 (1.2)	2	0 (0)	0	0 (0)	0	0 (0)	0	0 (0)	0
Cardiovascular events	0 (0)	0	0 (0)	0	1 (0.7)	1	0 (0)	0	0 (0)	0	0 (0)	0
Neoplasms	0 (0)	0	0 (0)	0	1 (0.7)	1	0 (0)	0	1 (1.0)	1	1 (1.5)	1
Psychiatric disorders	0 (0)	0	0 (0)	0	0 (0)	0	0 (0)	0	0 (0)	0	0 (0)	0
Immune-related events	0 (0)	0	0 (0)	0	0 (0)	0	0 (0)	0	0 (0)	0	0 (0)	0
GVHD	0 (0)	0	0 (0)	0	0 (0)	0	0 (0)	0	0 (0)	0	0 (0)	0

*One patient could have more that one AE episode

© 2025 Camacho-Arteaga L et al. *JAMA Network Open.*

eTable 7. All Infectious Episodes*

Infectious AEs	*Episodes*
SARS-CoV2 infection	45
Unspecified acute lower respiratory infection	14
Unspecified acute upper respiratory infection	12
Cystitis, unspecified	4
Enterocolitis due to Clostridium difficile	4
Gingivitis and periodontal diseases	4
Pneumonia without isolation	4
Urinary tract infection, site not specified	4
Herpes zoster	3
Influenza due to identified influenza virus	3
Pneumonia due to Pseudomonas	3
Campylobacter enteritis	3
Acute bronchitis due to respiratory syncytial virus	2
Candidal stomatitis	2
Diarrhoea and gastroenteritis of presumed infectious origin	2
Pneumonia due to Haemophilus influenzae	2
Pneumonia due to other infectious organisms, not elsewhere classified	2
Pseudomonas aeruginosa sepsis	2
Respiratory syncytial virus as the cause of diseases classified to other chapters	2
Septicaemia due to Klebsiella oxytoca	2
Actinomycosis, unspecified	1
Acute bronchitis due to other specified organisms	1
Acute bronchitis due to parainfluenza virus	1
Acute pharyngitis, unspecified	1
Acute tubulo-interstitial nephritis	1

© 2025 Camacho-Arteaga L et al. *JAMA Network Open.*

Acute upper respiratory infections of multiple and unspecified sites	1
Candidiasis	1
Catheter infection without isolation	1
Cholecystitis, unspecified	1
Cytomegaloviral disease	1
Escherichia coli in sputum	1
Haemophilus influenza in bronchoalveolar lavage	1
Herpes simplex infections	1
Infective spondylopathies without isolation	1
Influenza and pneumonia	1
Iridocyclitis, unspecified	1
Orchitis and epididymitis	1
Orchitis, epididymitis and epididymo-orchitis with abscess	1
Other interstitial pulmonary diseases with fibrosis	1
Otitis media, unspecified	1
Parvovirus infection, unspecified	1
Pilonidal cyst with abscess	1
Pneumocystosis	1
Pneumonia due to Acinetobacter	1
Salmonella enteritis	1
Scabies	1
Suppurative otitis media without isolation	1
**Total**	**146**

*A patient could have more than one episode

© 2025 Camacho-Arteaga L et al. *JAMA Network Open.*

eTable 8. Dermatologic Adverse Events

Dermatological AEs	***n***
Eczematous dermatoses	5
Eosinophilic dermatoses	5
Acne, unspecified	1
Actinic keratosis	1
Allergic urticaria	1
Benign lipomatous neoplasm	1
Erythema intertrigo	1
Erythema multiforme	1
Follicular disorder, unspecified	1
Other disorders of pigmentation	1
Postoperative seroma abdominal	1
Pruritus, unspecified	1
Psoriasis	1
Rash and other nonspecific skin eruption	1
Recurrent oral aphthae	1
Scabies	1
Secondary dermatitis due to malignant neoplasia	1
Spontaneous ecchymoses	1
Viral warts	1
**Total**	**27**

© 2025 Camacho-Arteaga L et al. *JAMA Network Open.*

eTable 9. Neurologic Adverse Events

Neurological AEs	***n***
Paresthesias	4
Unspecified encephalopathy	3
Polyneuropathy	1
Non-traumatic subdural hemorrhage	1
Intracerebral hemorrhage	1
Vocal cord paralysis	1
Tremor	1
Central vertigo	1
Postherpetic neuralgia	1
Bilateral psoas paresis	1
**Total**	**15**

© 2025 Camacho-Arteaga L et al. *JAMA Network Open.*

eTable 10. Cardiovascular Adverse Events

Cardiovascular AEs	***n***
Heart failure	6
Hypertrophic cardiomyopathy	1
Pericarditis	1
Syncope	1
Thoracic pain. unspecified	1
Interstitial fibrosis	1
Atrial fibrillation and flutter	1
Tachycardia. unspecified	1
**Total**	**13**

© 2025 Camacho-Arteaga L et al. *JAMA Network Open.*

eTable 11. Other Adverse Events

Other AEs	***n***
Functional diarrhoea	4
Abnormal laboratory results	3
Dyslipidemia	2
Embolism and thrombosis of unspecified vein	2
Benign neoplasm of colon, rectum, anus and anal canal	1
Bicipital tendinitis	1
Bronchitis, not specified as acute or chronic	1
Cholelithiasis	1
Fatty liver	1
Hypothyroidism	1
Idiopathic thrombocytopenic purpura	1
Interstitial pulmonary disease, unspecified	1
Lung diseases due to external agents	1
Magnesium deficiency	1
Other complications of internal prosthetic devices, implants and grafts, not elsewhere classified	1
Other diseases of lip and oral mucosa	1
Pain localized to upper abdomen	1
Postoperative intestinal obstruction	1
Pulmonary embolism	1
Rectal polyp	1
Renal failure	1
Tinnitus	1
Unilateral or unspecified inguinal hernia, with obstruction, without gangrene	1
**Total**	**30**

© 2025 Camacho-Arteaga L et al. *JAMA Network Open.*

eFigure. Cumulative Incidence of NRM

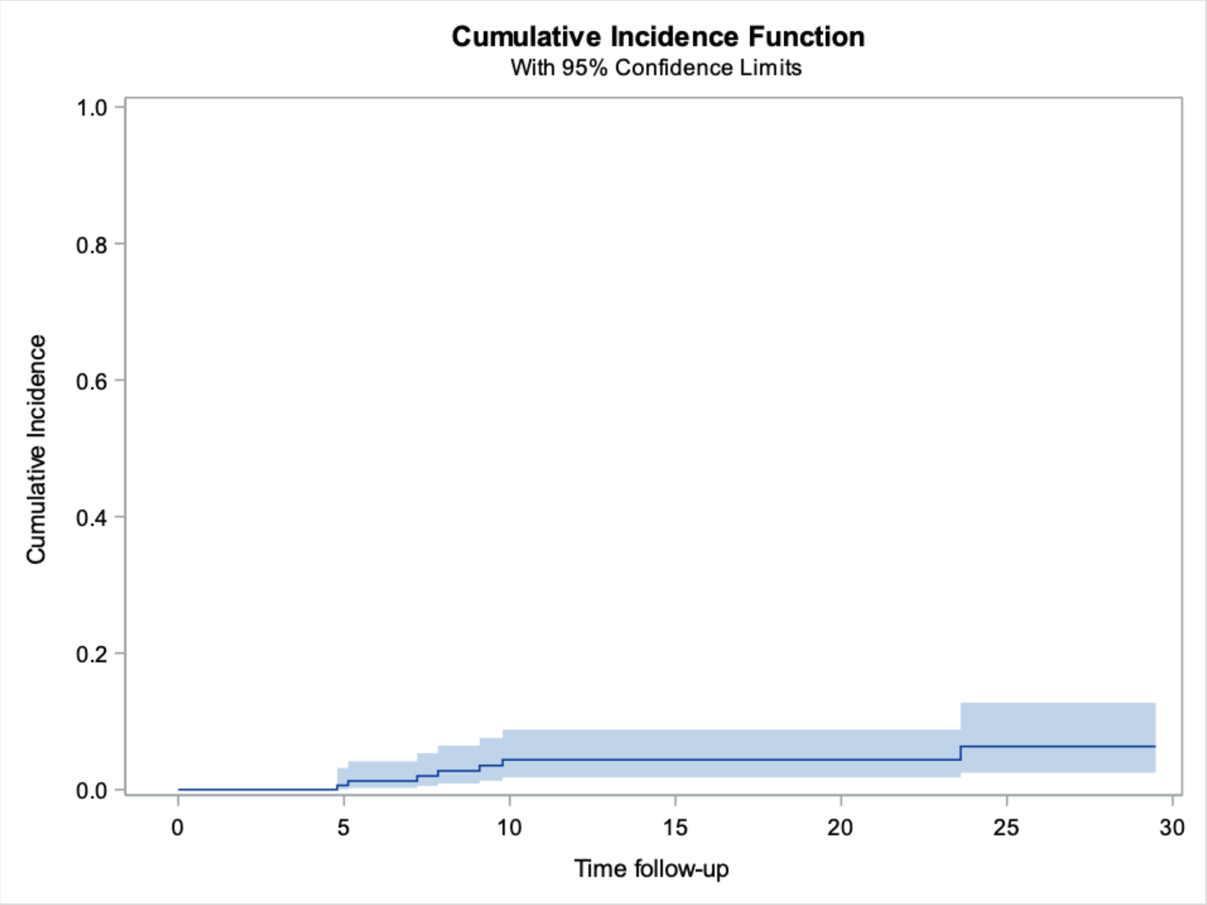

© 2025 Camacho-Arteaga L et al. *JAMA Network Open.*
