# Supplementary material for: Late Adverse Events After Chimeric Antigen Receptor T-Cell Therapy for Patients With Aggressive B-Cell Non-Hodgkin Lymphoma
Source: JAMA Netw Open. 2025 Feb 25;8(2):e2461683. doi: 10.1001/jamanetworkopen.2024.61683 (PMC11862965; doi:10.1001/jamanetworkopen.2024.61683)
Supplement: Supplement 2. — Data Sharing Statement [file jamanetwopen-e2461683-s002.pdf]

Data Sharing Statement

Camacho-Arteaga. Late Adverse Events After Chimeric Antigen Receptor T-Cell Therapy for Patients With Aggressive B-Cell Non-Hodgkin Lymphoma. *JAMA Netw Open*. Published February 25, 2025. doi:10.1001/jamanetworkopen.2024.61683

Data
Data available: Yes
Data types: Deidentified participant data
How to access data: Antonia.agusti@vallhebron.cat
When available: With publication

Supporting Documents
Document types: None

Additional Information
Who can access the data: esearchers whose proposed use of the data has been approved
Types of analyses: for a specified purpose
Mechanisms of data availability: with a signed data access agreement
